# Effect of Small‐Quantity Lipid‐Based Nutrient Supplementation on Children's Cortisol Concentration

**DOI:** 10.1111/mcn.70197

**Published:** 2026-05-21

**Authors:** Helena Nti, Paul D. Hastings, Seth Adu‐Afarwuah, Ebenezer Adjetey, Benjamin Amponsah, Charles D. Arnold, Lois M. D. Aryee, Fatimah B. Ayete Labi, Helena J. Bentil, Kathryn G. Dewey, Amanda E. Guyer, Adom Manu, Mavis O. Mensah, Elizabeth L. Prado, Elizabeth Shirtcliff, Xiuping Tan, Brietta M. Oaks

**Affiliations:** ^1^ Department of Nutrition and Food Science University of Ghana Accra Ghana; ^2^ Department of Nutrition and Dietetics University of Health and Allied Sciences Ho Ghana; ^3^ Center for Mind and Brain University of California Davis Davis California USA; ^4^ Department of Psychology University of California Davis Davis California USA; ^5^ Department of Psychology University of Ghana Accra Ghana; ^6^ Institute for Global Nutrition University of California Davis Davis California USA; ^7^ Department of Human Ecology University of California Davis Davis California USA; ^8^ School of Public Health University of Ghana Accra Ghana; ^9^ Department of Psychiatry Harvard Medical School Boston Massachusetts USA; ^10^ Department of Nutrition University of Rhode Island Kingston Rhode Island USA

**Keywords:** adolescents, first 1000 days, Ghana, hair cortisol concentration, hypothalamic‐pituitary adrenal axis, SQ‐LNS

## Abstract

This study investigated the role of early‐life nutrition supplementation in regulating the development of hypothalamic‐pituitary‐adrenal (HPA) axis activity, as evidenced by hair cortisol concentration (HCC), in the iLiNS‐DYAD randomized controlled trial in Ghana. Pregnant women were randomized to one of three conditions: (1) iron and folic acid (IFA) during pregnancy and placebo 0‐6 mo postpartum; (2) multiple micronutrients (MMN) during pregnancy and 0–6 mo postpartum; or (3) small‐quantity lipid‐based nutrient supplements (SQ‐LNS) during pregnancy and 0–6 mo postpartum and for their children from 6 to 18 mo. At 9–11 y of age, usable hair samples were obtained from 680 children from which cortisol was assayed. ANCOVA models assessed differences between groups and potential effect modifiers, including maternal education, household asset index, pre‐pregnancy BMI, child sex, child BMI, and pubertal stage. HCC did not differ between SQ‐LNS and control groups in adjusted and unadjusted models (*p* > 0.10), but maternal education was a significant effect modifier (*P*‐interaction = 0.043). Children exposed to SQ‐LNS had lower HCC than control children among those whose mothers had 0–5 years schooling (Median (interquartile range): 6.3 (4.8, 9.9) vs. 7.8 (5.3, 9.9); *p* = 0.031) but not among those whose mothers had > 5 years of schooling (*p* = 0.949). No other interactions were significant. Although these findings should be viewed with caution, they suggest that early‐life SQ‐LNS may buffer physiological stress in children of less educated mothers, highlighting its potential to compensate for socioeconomic disadvantage with regard to HPA axis regulation.

**Trial Registration:** Clinical Trial Registry number and website: clinicaltrials.gov as NCT00970866 https://clinicaltrials.gov/ct2/show/record/NCT00970866.

## Summary

- No difference in hair cortisol concentration at ages 9–11 between children in the early life SQ‐LNS and control groups.
- Among children of parents with 0–5 years of schooling SQ‐LNS was associated with lower cortisol concentrations compared to controls.
- No effect modification by maternal education was observed among children whose mothers had > 5 years of schooling.

**Abbreviations**
HCC: hair cortisol concentration
HPG: hypothalamic pituitary gonadal
IFA: iron and folic acid
iLiNS: International lipid based nutrient supplements
MMN: multiple micronutrients
RCT: randomized controlled trial
SES: socioeconomic status
SQ‐LNS: small‐quantity lipid‐based nutrient supplements

## 1 Introduction

Early life nutrition plays a critical role in shaping an individual's long‐term health and development. During the first 1000 days of life, spanning from conception to a child's second birthday, the foundations for cognitive, physical, and emotional health are established (Cusick and Georgieff 2022; Prado and Dewey 2014). Adequate nutrition during this period is essential for optimal growth, immune function, and neurodevelopment (Cusick and Georgieff 2022; Fuglestad et al. 2008). However, many children under 5 years of age in low‐ and middle‐income countries (LMICS) suffer from malnutrition, which can lead to adverse health outcomes later in life (Victora et al. 2021). One approach to preventing early malnutrition has been through the use of small‐quantity lipid‐based nutrient supplements (SQ‐LNS), which are designed to provide essential micronutrients and fatty acids, improving overall dietary quality and reducing stunting, wasting, mortality, anemia, and impaired development among children 6–23 months of age (Dewey et al. 2021).

Cortisol is a glucocorticoid hormone (Noppe et al. 2014a) released by the hypothalamic‐pituitary adrenal (HPA) axis (Wagner et al. 2019) in response to exposure to stressful experiences. It plays a pivotal role in the body's stress response, influencing metabolic processes and immune function. Cortisol can be measured in various bodily fluids, including serum, saliva, and urine, but these are influenced by circadian rhythm, pulsatile secretion, and acute stress, complicating the interpretation of these biomarkers (Noppe et al. 2014a). Hair cortisol concentration (HCC) has emerged as a valuable biomarker of long‐term HPA axis activity indicative of systemic stress exposure, providing a retrospective index of cumulative cortisol secretion over extended periods, typically up to 6 months (Wagner et al. 2019; Groeneveld et al. 2013; Wester and van Rossum 2015; Faresjö et al. 2023). Various factors are associated with higher HCC, including higher body mass index (BMI) (Noppe et al. 2014a), lower socioeconomic status (SES) (Gray et al. 2018), and being male during childhood (Gray et al. 2018), although sex differences are reduced during adolescence (Adu‐Afarwuah et al. 2015; Bentil et al. 2024; Nti et al. 2024). Lower maternal education and SES have been associated with elevated stress markers in children (Adu‐Afarwuah et al. 2015; Bentil et al. 2024; Nti et al. 2024; Lupien et al. 2000; Vliegenthart et al. 2016). The HPA axis undergoes reactivation during puberty, potentially making this developmental window particularly sensitive to both current stressors and the effects of earlier exposures (Kang et al. 2014; Moskow et al. 2016).

Early‐life malnutrition adversely affects the development of multiple essential physiological systems (Forgie et al. 2020; Campisano et al. 2019; Kaput et al. 2014). Suboptimal nutrition during the first 1000 days of life may impair the proper regulation of the HPA axis both in early life and subsequently, including during early puberty, potentially leading to altered cortisol production and stress reactivity later in life (Maniam et al. 2014). Thus, adequate nutrition during early life may be essential for promoting healthy development of the HPA axis (Hoeijmakers et al. 2015; Cohen Kadosh et al. 2021). SQ‐LNS are formulated to provide essential vitamins and minerals that may be deficient in the diets of pregnant women and young children in resource‐poor settings. These supplements are typically composed of a lipid base (omega‐3 rich vegetable oil and a legume such as peanut butter) enriched with milk powder and essential micronutrients.

Several micronutrients included in SQ‐LNS, such as iron, zinc, folate, and omega‐3 fatty acids, play essential roles in the development and regulation of the HPA axis (Prado and Dewey 2014; Mansour 2022). Iron and zinc are involved in neurotransmitter synthesis and receptor function, which are critical for proper stress hormone signaling (Prado and Dewey 2014; Prakash et al. 2015). Folate supports methylation processes important for gene regulation within stress‐related brain circuits, while omega‐3 fatty acids contribute to neuronal membrane integrity and anti‐inflammatory responses that influence HPA activity (Zinkow et al. 2024; Bekdash 2023). Deficiencies in these nutrients during critical windows of early development may impair the structural and functional maturation of brain regions such as the hippocampus and amygdala, leading to dysregulated cortisol production and heightened stress sensitivity (Yam et al. 2015). Thus, adequate nutrient intake in early life is crucial for programming a well‐regulated HPA axis and adaptive stress responses—likely buffering these stress‐related effects, especially in disadvantaged populations (Dewey et al. 2022). Despite this biological plausibility, the effect of early nutritional supplementation on HCC in children and youths remains understudied.

The International Lipid‐Based Nutrient Supplements (iLiNS) DYAD trial in Ghana was a three‐arm randomized controlled trial (RCT) designed to evaluate the effects of prenatal and early childhood SQ‐LNS in comparison with control groups in which the mothers received either iron and folic acid (IFA) or multiple micronutrients (MMN) but their children did not receive any supplementation. We previously found no significant difference in HCC between the SQ‐LNS and IFA groups at ages 4–6 years (IFA: 5.37 pg/mg, SQ‐LNS: 4.95 pg/mg; *p* = 0.290) (Oaks et al. 2020). However, the HPA axis is reactivated at puberty and thus effects of early life nutrition may be more apparent in older children. Therefore, our primary objective herein was to assess the effect of SQ‐LNS consumed by mothers during pregnancy and lactation, as well as by their children from 6 to 18 months of age, on the children's HCC at 9–11 years of age. We hypothesized that children in the SQ‐LNS group would exhibit lower HCC than children in the IFA and MMN groups. Because effect modification by child sex and maternal or household characteristics has been observed for other outcomes in the iLiNS‐DYAD trial (Adu‐Afarwuah et al. 2019; Prado et al. 2023) we tested for effect modification by six variables: child sex, child body mass index for age z‐score (BMIZ), pubertal stage, asset index, maternal education and pre‐pregnancy BMI. We did not set a priori hypotheses for the effect modification analyses.

## 2 Methods

### 2.1 Participants and Procedure

Details of the ILiNS‐DYAD–Ghana trial, performed in 2009–2014 (Adu‐Afarwuah et al. 2015, 2016), and the 4–6 year follow‐up study performed in 2016 (Ocansey et al. 2019; Kumordzie et al. 2019), have been previously reported and are summarized herein. This was a partially double‐blind RCT that evaluated the effects of three different nutrient supplements. The study took place in peri‐urban areas within the Yilo Krobo and Lower Manya Krobo districts in Ghana's Eastern Region, approximately 70 km north of Accra, the capital. Written informed consent was obtained from primary caregivers prior to collecting data. Data collection involved both home visits and office appointments, where socio‐demographic information was gathered through interviewer‐administered questionnaires.

Women were eligible for participation if they were 18 years of age or older and less than 20 weeks of gestation. Exclusion criteria included the following: indication of HIV infection, asthma, epilepsy, tuberculosis, or any malignancy on the antenatal card; known allergies to milk or peanuts; not residing in the study area; planning to relocate within the next 2 years; unwillingness to consent to participate, receive home visits from fieldworkers, or take the study supplement; and involvement in another trial.

Pregnant women attending antenatal clinics in four main health facilities in the study area were recruited from December 2009 to December 2011. Eligible women were visited at home and those who met the inclusion criteria and provided informed consent were scheduled for a clinic visit for baseline assessments.

Enrolled women were randomly assigned to one of three supplementation groups (Table 1): (i) Daily 60 mg iron and 400 mg folic acid (IFA) during pregnancy, and 200 mg calcium only (as a placebo) during the first 6 mo postpartum, with no supplementation for offspring; (ii) Daily multiple micronutrients (MMN; 1‐2 RDA of 18 vitamins and minerals) during pregnancy and the first 6 mo postpartum, with no supplementation for offspring, and (iii) Daily 20 g SQ‐LNS during pregnancy and the first 6 mo postpartum with daily 20 g SQ‐LNS for offspring from 6 to 18 mo of age.

**Table 1:** Nutrient and energy contents of supplements used in the iLiNS trial in Ghana.

	IFA	MMN	Maternal SQ‐LNS	Child SQ‐LNS
Ration per day	1 capsule	1 capsule	20‐g sachet	20‐g sachet
Total energy, kcal	0	0	118	118
Protein, g	0	0	2.6	2.6
Fat, g	0	0	10	9.6
Linoleic acid, g	0	0	4.59	4.46
α‐Linolenic acid, g	0	0	0.59	0.58
Vitamin A, μg RE	0	800	800	400
Vitamin C, mg	0	100	100	30
Vitamin B‐1, mg	0	2.8	2.8	0.3
Vitamin B‐2, mg	0	2.8	2.8	0.4
Niacin, mg	0	36	36	4
Folic acid, μg	400	400	400	80
Pantothenic acid, mg	0	7	7	1.8
Vitamin B‐6, mg	0	3.8	3.8	0.3
Vitamin B‐12, μg	0	5.2	5.2	0.5
Vitamin D, mg	0	10	10	5
Vitamin E, mg	0	20	20	6
Vitamin K, μg	0	45	45	30
Iron, mg	60	20	20	6
Zinc, mg	0	30	30	8
Copper, mg	0	4	4	0.34
Calcium, mg	0	0	280	280
Phosphorus, mg	0	0	190	190
Potassium, mg	0	0	200	200
Magnesium, mg	0	0	65	40
Selenium, μg	0	130	130	20
Iodine, μg	0	250	250	90
Manganese, mg	0	2.6	2.6	1.2

Abbreviations: IFA, iron and folic acid capsule; MMN, multiple micronutrient supplement capsule; SQ‐LNS, small‐quantity lipid‐based nutrient supplement. Information from table previously published (Prado et al. 2016).

### 2.2 Data Collection at Enrollment

At enrollment during the main trial, we determined maternal baseline characteristics including age, education, marital status, depressive symptoms (using the Center for Epidemiological Studies–Depression test (Radloff 1977)), weight (Seca 874; Seca), height (Seca 217; Seca), hemoglobin concentration (Hb; HemoCueAG), pre‐pregnancy BMI, household asset index (proxy indicator for household SES constructed for each household based on ownership of a set of assets (Prado et al. 2023)), household food security (HFIAS, Household Food Insecurity Access Scale (Coates et al. 2007)), home environment (Home Observation for the Measurement of the Environment Inventory (Caldwell and Bradley 1984)), gestational age (by ultrasound) and parity (Adu‐Afarwuah et al. 2015). Birth weight (BW) was assessed to the nearest 20 g (Seca 383; Seca), within 48 h after birth or between 3 and 14 days after birth for 87 (9.4%) infants when measurement within 48 h was not feasible (Prado et al. 2023).

### 2.3 First Follow‐Up Study

The first follow‐up study was conducted in 2016 when children were 4–6 years of age. Child BMI was assessed (Kumordzie et al. 2019). Other procedures and measures were not pertinent to the current analyses, but have been reported previously.

### 2.4 Follow‐Up at 9–11 Years of Age (2020–2021)

Caregivers of surviving children from the original iLiNS‐DYAD‐Ghana trial were located using previously recorded addresses and phone numbers and were invited to join this follow‐up study. Ethical approval for the study was granted by the Institutional Review Board at the University of California‐Davis (IRB ID: 1489918) and the Ghana Health Service Ethical Review Committee (GHS‐ERC: 027105119). Written informed assent was obtained from each child before data collection began. During their visit to our project office, hair samples were collected from each child for cortisol analysis.

Child weight was measured to the nearest 50 g (Seca 875 scale) and height was measured using a stadiometer (Seca 217) to 0.1 cm. All measurements were taken twice, and a third measurement was taken if the initial two readings differed by more than 0.1 kg for weight or 0.5 cm for height. Body mass index was standardized as z‐score (BMIZ) using WHO norms.

Pubertal status was assessed using the Petersen Pubertal Development Scale (PDS) (Petersen et al. 1988). Participants rated each of the five pubertal milestones for boys and girls on a 4‐point Likert scale, where 1 indicated that pubertal development had not yet begun, 2 indicated that it had barely started, 3 indicated that it was definitely underway, and 4 indicated that it was completed. In girls, the PDS score was determined by self‐reports on breast development, menarche, growth spurt, skin changes, and body hair. For boys, pubertal development was assessed based on self‐reports of voice changes, facial hair, growth spurt, skin changes, and body hair. The total PDS score ranged from 5 to 20.

Hair samples were collected from the posterior vertex, close to the scalp. Each sample was approximately 3–5 mm in length, weighing ~100 mg, and comprised 100–120 strands. The scalp end was secured with a rubber band, and the sample was wrapped in foil, stored at room temperature, and shipped to the Stress Physiology Investigative Team (SPIT) laboratory at the University of Oregon for cortisol analysis.

### 2.5 Assessment of Hair Cortisol

At the SPIT laboratory, hair samples (cut if necessary to usable part from the scalp) were collected into test tubes, and washed twice in high performance liquid chromatography (HPLC)‐ grade isopropanol with 3 min (min) of inversion on a rotator per wash. The cleaned hair was dried with forced air for 4–5 h and left overnight to ensure complete evaporation of isopropanol and dryness.

A 15 mg portion of cleaned hair was placed in a 2 ml microcentrifuge tube along with three 4.9 mm stainless steel beads. The hair was finely ground using a Retsch ball mill (MM400) at 30 Hz for 8 min. For the extraction process, 1.5 mL of HPLC‐grade methanol was added, and the samples were incubated for 24 h at room temperature with continuous inversion on a rotator. Following extraction, the samples were centrifuged, and 1 mL of supernatant was moved to a fresh microcentrifuge tube.

The methanol was then evaporated under a nitrogen stream at 50°C for 15–20 min. The steroid extract was reconstituted with 150 μL of assay diluent, vortexed for 30 s to 1 min, and immediately analyzed using a commercially available enzyme‐linked immunosorbent assay (ELISA) kit (Salimetrics, State College, PA, USA). This ELISA kit was validated for analyzing hair cortisol, with an assay sensitivity of 1.05 pg/mg. Each sample was analyzed in duplicate, and the intra‐ and inter‐assay coefficients of variation were maintained below the 10% cutoff for absorbance at 450 nm.

Cortisol concentration was determined by comparing absorbance values with the standard curve generated from known cortisol concentrations. The concentration obtained (pg/mL) was adjusted for the extraction volume (1.5 mL) and normalized to the hair sample weight (15 mg) to yield a final cortisol concentration expressed in pg of cortisol per mg of hair (pg/mg).

### 2.6 Statistical Analysis

Analyses were conducted using R version 4.3.1 (2023‐06‐16 ucrt, R Foundation for Statistical Computing, Vienna, Austria). This was performed on 680 children who consented and were available for the collection of hair samples, providing 80% power to detect an SQ‐LNS effect size of 0.46 SD or greater. We posted a statistical analysis plan to Open Science Framework (https://osf.io/jw2tc) before conducting analyses. We conducted a complete case intention‐to‐treat analysis.

We performed a pre‐specified sensitivity analysis to compare potential differences in HCC between the IFA and MMN groups at a significance threshold of *p* < 0.10. As these two groups were similar (*p* = 0.419), subsequent analyses were performed comparing the SQ‐LNS group with the combined non‐LNS (IFA and MMN) groups. Nevertheless, results from the three‐group analysis are also presented to illustrate differences across the intervention arms (see Supporting Information S1: Table 1). The HCC was log transformed (logHCC) to satisfy model assumptions. Geometric mean ratios were tested using regression analysis, with statistical significance set at *p* < 0.05.

We assessed two models: (1) Model 1, adjusted for child age at 9–11 years, and (2) Model 2, adjusted for child age at 9–11 years, child sex, and pubertal stage. Pre‐specified covariates identified from previous studies (Wester and van Rossum 2015; Gray et al. 2018; Vliegenthart et al. 2016; Oaks et al. 2020) included baseline and follow‐up characteristics: household asset, household food insecurity, home environment, years of maternal education, pre‐pregnancy BMI, maternal depressive symptoms, birth order, age and sex; birth weight, child BMI at 4–6 years/9–11 years and season of hair sample collection. In separate models, we evaluated effect modification by household asset index, child BMI, maternal education, pubertal stage, pre‐pregnancy BMI and child sex.

Maternal education was subsequently analyzed as a dichotomous variable to facilitate the interpretation of interaction effects and improve statistical power. A cut‐off of 5 years was used based on educational milestones in the study setting, where completion of elementary school typically corresponds to at least 5 years of formal education. Mothers with 0‐5 years of schooling were categorized as having lower education, while those with more than 5 years were considered to have higher education.

## 3 Results

**Figure 1:**
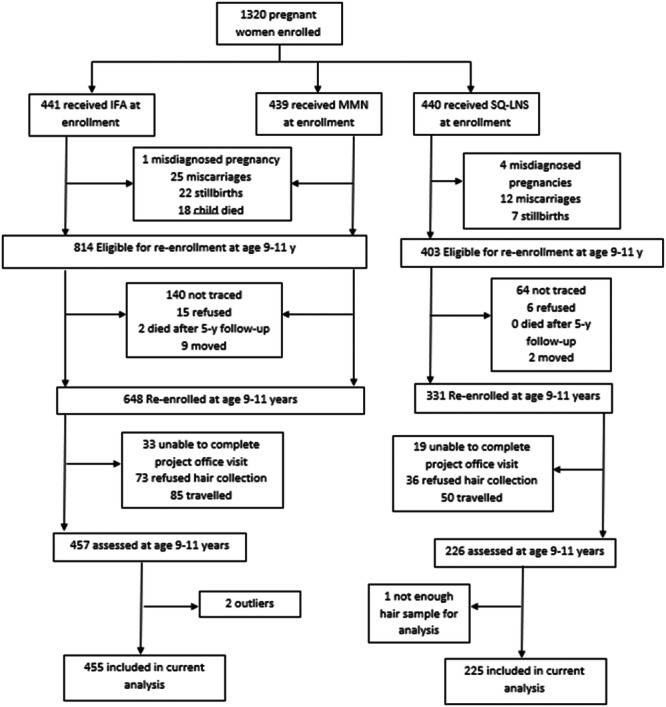
Flowchart of child's eligibility, enrollment and data collection at 9–11 y when we assessed hair cortisol concentration. IFA = Iron & folic acid supplement, MMN = Multiple micronutrient supplement, SQ‐LNS = Small‐quantity lipid‐based nutrient supplement.

Of the 1217 children eligible to participate in the follow‐up study, we re‐enrolled 979 at 9–11 years of age and assessed 683 children who were available and consented to HCC assessment (Figure 1). Two children had highly elevated HCC levels that were outliers, whereas too little hair was obtained from one child to analyze; these three children were excluded. The current analyses included 225 and 455 children in the SQ‐LNS and non‐LNS groups, respectively. The maternal baseline characteristics of this sample are presented in Table 2.

**Table 2:** Selected maternal baseline characteristics.

Variable	SQ‐LNS (*n* = 225) Mean (SD) or % (*n*/total)	Non‐LNS (*n* = 455) Mean (SD) or % (*n*/total)
Age, y	26.9 (5.4)	27.1 (5.4)
Education, y	7.7 (3.8)	7.6 (3.2)
Married or cohabiting, % (*n*/*N*)	92.9 (209/2)	94.9 (432/455)
Maternal depressive symptoms	4.6 (4.4)	5.4 (4.8)
Household asset index z‐score^a^	−0.08 (1.01)	0.06 (1.00)
Household food secure, % (*n*/*N*)	62.2 (140/224)	58.0 (264/451)
Household improved water source, % (*n*/*N*)	99.1 (223/225)	98.5 (448/455)
Household toilet facility, % (*n*/*N*)	98.2 (221/225)	97.6 (444/455)
Height, cm	159.2 (5.4)	158.8 (5.9)
Pre‐pregnancy BMI^b^, kg/m^2^	24.8 (4.6)	24.3 (4.6)
Hemoglobin concentration, g/L	111.8 (11.2)	111.7 (12.6)
Primiparous, % (*n*/N)	30.7 (69/225)	28.8 (131/455)

Abbreviations: Non‐LNS = Iron & folic acid + multiple micronutrient groups, SQ‐LNS = small quantity lipid‐based nutrient supplement.

^a^ Proxy indicator for household socioeconomic status constructed for each household based on ownership of a set of assets (radio, television etc.), lighting source, drinking water supply, sanitation facilities, and flooring materials. Household ownership of this set of assets is combined into an index (with a mean of zero and standard deviation of one) using principal components analysis. Higher value represents higher socioeconomic status.

^b^ Estimated pre‐pregnancy BMI was calculated from estimated pre‐pregnancy weight (based on polynomial regression with gestational age, gestational age squared, and gestational age cubed as predictors) (Adu‐Afarwuah et al. 2017) and height at enrollment.

The 680 children included in the current analysis were similar to the 638 children who had missing HCC values in most background characteristics such as baseline food security, asset index and maternal education. However, they differed in child sex, baseline maternal age, mother married/cohabiting and parity (included as covariates) (see Supporting Information S1: Table 2); children with HCC values were more likely to be female (*p* = 0.083), and to have mothers who were married/cohabiting (*p* = 0.043), older (*p* = 0.042), and had given birth to one or more prior children (*p* = 0.001), compared to those without HCC values.

**Figure 2:**
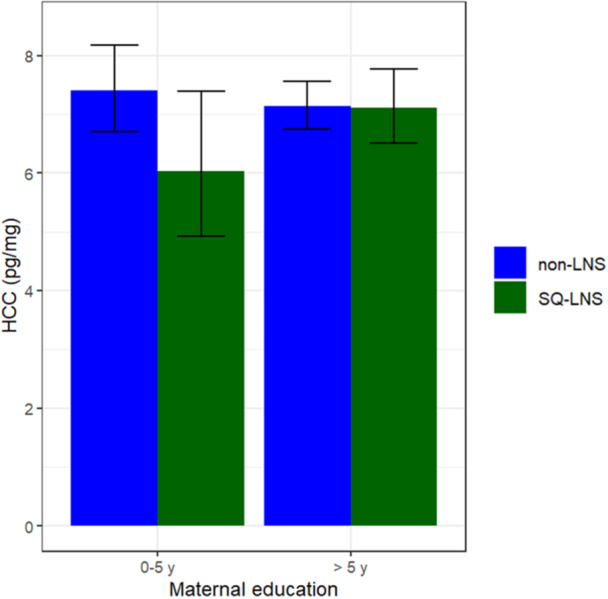
Intervention effect modification by baseline maternal education on log‐transformed hair cortisol concentration (logHCC). SQ‐LNS = Small‐quantity lipid‐based nutrient supplement. non‐LNS = Iron & folic acid + multiple micronutrient supplement groups. *n* (%): SQ‐LNS: 0‐5 y = 49 (7%), > 5 y = 176 (26%); non‐LNS: 0‐5 y = 91 (13%), > 5 y = 364 (54%).

Children included in our study had normal average HCC at 9–11 years of age, based on reference values for healthy children (6.8–8.5 pg/mg (Noppe et al. 2014b)). Median (interquartile range) pg/mg HCC did not differ between the SQ‐LNS (7.4 (5.0, 10.1)) and non‐LNS groups (7.5 (5.4, 10.2)) in either unadjusted or adjusted models (*p* > 0.10). Among the six interaction effect analyses, maternal education modified the intervention effect (*P*‐interaction = 0.043) on HCC, with significant group differences in HCC among children of mothers with 0–5 years of education (SQ‐LNS 6.3 (4.8, 9.9) vs non‐LNS 7.8 (5.3, 9.9), *p* = 0.031) but not among those whose mothers had > 5 years (*p* = 0.949) (Figure 2). No other interactions were significant.

## 4 Discussion

To our knowledge, this is the first study to examine the impact of SQ‐LNS provided during pregnancy and early childhood on stress exposure as measured by hair cortisol concentration in late childhood. We found that SQ‐LNS did not significantly impact HCC in the full sample, which does not support our hypothesis that the SQ‐LNS group would have lower HCC than the non‐LNS group. Similarly, our previous research also did not find evidence of an intervention effect on HCC at 4–6 years of age (Oaks et al. 2020). However, in the current study, we found that among children of mothers who had 0–5 years of education, HCC was significantly lower in the SQ‐LNS group than in the non‐LNS group, whereas there was no group difference among children whose for mothers who had > 5 years of education. Because maternal education was one of six different potential effect modifiers examined and the other five did not exhibit interaction effects, this finding could be due to chance and should be interpreted with caution, providing justification for further research but not definitive conclusions.

Nonetheless, the effects of SQ‐LNS on HCC among children of mothers with less education are plausible, given that such children were likely at higher risk of early‐life nutritional deficiencies, which could affect the HPA‐axis regulation. SQ‐LNS provides key micronutrients like zinc, iron and omega‐3 fatty acids that are critical for brain development and stress regulation (Shi et al. 2025; Oravcova et al. 2022). In our previous publication, the provision of SQ‐LNS during the first 1000 days improved child behavior (at 4–6 years), particularly for children from low nurturing and stimulation households (Ocansey et al. 2019). Mothers with lower education levels, which often co‐occurs with lower resources in the household, may experience hardship, leading to altered parenting styles, stress regulation and home environments that shape children's physiological stress responses (Christensen et al. 2024; Scattolin et al. 2022). While such conditions may increase stress exposure, some studies suggest that children in lower SES households may develop adaptive stress regulation strategies, particularly if their environments provide support or resilience‐building experiences (Graber and Kara 2021). Conversely, maternal education also influences language exposure and emotional regulation strategies (Cuartas 2022; Huang et al. 2022; Britto et al. 2006). Mothers with higher education are more likely to engage in more cognitive stimulating interactions which can shape children's stress responses.

Few studies have examined how biomarkers of the HPA axis in children respond to nutritional or health interventions. Lin et al. (2021) conducted an early life cluster randomized controlled trial in Bangladesh involving both a nutrition intervention and a water, sanitation and handwashing intervention, and measured salivary cortisol reactivity to a stressor at 2 years (Lin et al. 2021). Toddlers who had received the combined environmental and nutritional interventions exhibited greater cortisol reactivity compared to those in the control group. However, salivary cortisol reflects acute stress responses, whereas HCC reflects chronic stress activation, and there is maturation of the HPA axis from the toddler to pre‐adolescent periods. Therefore, the findings of Lin et al. cannot be compared directly to the current findings. Notably, long‐term effects are subtle, and our study likely was not sufficiently powered to detect them.

Additionally, it is possible that in early life, there may be compensatory mechanisms that maintain the HPA axis function within a normal range, despite variations in nutritional intake (Barker 1998; Wells 2007). These mechanisms could buffer against the impact of the intervention. Alternatively, it is conceivable that the effect of SQ‐LNS on the activity of the HPA axis may not present as changes in HCC in early puberty, but may become more evident at mid and late puberty when cortisol concentrations are higher (Blumenthal et al. 2014; King et al. 2017).

Our study has several strengths. First, we assessed hair cortisol concentration which represents a retrospective assessment of medium and long‐term cortisol concentration. Second, children in the SQ‐LNS and non‐LNS groups were similar in baseline characteristics except for food security and asset index, which means that our sample remained balanced over time. One limitation of our study was that children who were included in the current study differed from those not included in key characteristics, including baseline maternal age, marital/cohabiting status, parity and child sex (*p* < 0.10). These differences may affect the generalizability of our findings to the entire sample. In addition, we did not apply corrections for multiple hypothesis testing in the effect modification analyses. Although the effect modifiers were pre‐specified, such analyses are inherently exploratory, and we therefore did not adjust for multiple comparisons. Nonetheless, since the p‐for‐interaction was significant for only one of the pre‐specified effect modifiers, the observed effects among children of mothers with lower education should be interpreted with caution, as they may be due to chance.

In conclusion, our study did not show a main effect of early life nutrition intervention on children's stress exposure as measured by hair cortisol concentration examined at 9–11 years of age. However, our results suggest that an effect of SQ‐LNS on lowering cortisol at early puberty was evident among children of mothers with lower education, suggesting a potential buffering role against socioeconomic disadvantage in HPA axis regulation. Future research should focus on whether these differences in this marker of stress exposure persist into later ages, particularly among more vulnerable children.

## Author Contributions

H.N., B.M.O., S.A.A., E.L.P., P.D.H., A.E.G., K.G.D., A.M. and B.A. conceptualized and designed the follow‐up study; H.N., S.A.A., E.L.P., E.A., H.J.B., M.O.M. and X.T. conducted the research; H.N. and C.A. conducted the statistical analyses; H.N. led the writing of the manuscript with critical review from B.M.O., C.D.A., P.D.H., A.E.G., K.G.D., S.A.A., H.J.B., L.M.D.A., M.O.M. and F.A.L., H.N. and B.M.O. had primary responsibility for final content. Due to her death during the preparation of this manuscript co‐author E.L.P. was unable to read and approve the final manuscript.

## Conflicts of Interest

The authors declare no conflicts of interest.

## Supporting information

Supporting File

## Data Availability

Data on de‐identified individuals, along with the code book and analytical code, will be accessible to researchers upon request. Access is contingent on the submission of a methodologically robust proposal and statistical analysis plan, subject to approval by the Principal Investigators. Proposals should be directed to the corresponding author.
