# Supplementary material for: Effect of Small‐Quantity Lipid‐Based Nutrient Supplementation on Children's Cortisol Concentration
Source: Matern Child Nutr. 2026 May 21;22(3):e70197. doi: 10.1111/mcn.70197 (PMC13205876; doi:10.1111/mcn.70197)
Supplement: Supplementary file 1 — Supporting File [file MCN-22-e70197-s001.docx]

Supplementary Table 1 Comparison of Hair Cortisol Concentrations Across SQ-LNS, MMN and IFA Intervention Arms

| Median  (Interquartile Range) HCC (pg/mg) | SQ-LNS | MMN | IFA | Unadjusted  *P* value  IFA vs SQ-LNS | Unadjusted  *P* value  IFA vs MMN | Adjusted  *P* value  IFA vs SQ-LNS | Adjusted  *P* value  IFA vs MMN |
| --- | --- | --- | --- | --- | --- | --- | --- |
| 9-11 y | 7.4 (5.0, 10.1) | 7.8 (5.7, 10.4) | 7.1 (5.1, 10.1) | 0.627 | 0.446 | 0.555 | 0.512 |

The SQ-LNS, MMN and IFA groups did not differ in both unadjusted and adjusted models
